# A Prospective Comparison of Ultrasound‐Guided Ankle Versus Popliteal Nerve Blocks for Elective Forefoot Surgery in a UK Podiatric Surgery Unit

**DOI:** 10.1002/jfa2.70219

**Published:** 2026-09-23

**Authors:** Yang Sern Kang, Tommy Chan, Robert Morley

**Affiliations:** ^1^ Surgical Trainee in Podiatric Surgery Department of Podiatric Surgery Derbyshire Community Health Services NHS Foundation Trust Ilkeston Derbyshire UK; ^2^ Consultant Podiatric Surgeon Department of Podiatric Surgery Derbyshire Community Health Services NHS Foundation Trust Ilkeston Derbyshire UK; ^3^ Consultant Podiatric Surgeon Department of Podiatric Surgery Doncaster & Bassetlaw Hospitals NHS Foundation Trust Mexborough South Yorkshire UK; ^4^ Consultant Podiatric Surgeon Coriel Orthopaedic Group Doncaster UK; ^5^ Consultant Podiatric Surgeon Independent Health Group Bath UK

## Abstract

**Background:**

Regional anaesthesia is central to perioperative care in foot surgery, yet comparative data within UK podiatric surgery services remain limited. Establishing local outcome baselines is essential for optimising care and supporting clinical scope.

**Aim:**

To compare pain intensity levels and functional recovery in patients undergoing elective forefoot surgery under ultrasound‐guided ankle or popliteal nerve block.

**Methods:**

This prospective service evaluation involved 17 adults undergoing elective forefoot surgery. Participants were allocated via pragmatic, nonrandomised sequential scheduling based on surgeon preference to receive either an ultrasound‐guided ankle block (*n* = 9) or popliteal nerve block (*n* = 8) using 0.5% levobupivacaine. Outcomes included sensory onset time, duration of analgesia, pain intensity (VAS), opioid consumption standardised to morphine milligram equivalents (MME), and functional recovery assessed using a modified Functional Recovery Index (FRI).

**Results:**

Sensory onset times showed no significant difference between groups (*p* = 0.491). Popliteal blocks provided significantly longer analgesia than ankle blocks (23.75 vs. 12.61 hours; *p* = 0.038), whereas procedural discomfort was comparable (*p* = 0.449). Postoperative pain trajectories differed significantly (*p* = 0.049), with pain decreasing from Day 1 to Day 3 following ankle block but increasing following popliteal block. Opioid consumption declined over time across the cohort (*p* = 0.015), with no significant between‐group differences. Functional recovery demonstrated a trend towards greater early improvement in the ankle group.

**Conclusions:**

Both ankle and popliteal blocks provided effective perioperative anaesthesia for elective forefoot surgery. Popliteal blocks offered longer analgesia, whereas ankle blocks showed a trend towards earlier mobilisation. Although limited by sample size and Type II error risk, these findings provide baseline data to inform future adequately powered studies and contribute evidence from UK podiatric day‐case practice that may have relevance to wider day‐case foot surgery.

## 1 Introduction and Background

Forefoot surgeries, including hallux abducto valgus (bunion) correction, lesser toe deformity correction, and metatarsal osteotomies, are the most frequently performed elective foot procedures [1, 2, 3]. These operations are increasingly being undertaken as day‐case procedures under local anaesthesia alone, without general anaesthesia or intravenous sedation, reflecting contemporary trends in ambulatory surgical practice [4, 5]. This approach aligns with the growing policy emphasis on enhanced recovery, day‐case pathways, and minimised opioid use, all of which are central principles of Enhanced Recovery After Surgery (ERAS) programmes and the Getting It Right First Time (GIRFT) initiative [6, 7, 8]. Consequently, effective regional anaesthesia has become fundamental to optimising perioperative pain control, patient recovery, and day‐case surgical efficiency.

Podiatric surgery occupies a distinct role within the United Kingdom (UK), with regional local anaesthesia typically delivered by specialised podiatrists within the surgical team. The reliable and adequate pain control afforded using these regional techniques enabled the widespread emergence and growth of day‐case foot surgery under local anaesthesia. Two predominant options for forefoot surgery are the ankle and popliteal nerve block, with each technique carrying particular anatomical implications, performance logistics, analgesia, motor‐sensory trade‐offs, and potential implications for service delivery. Ankle block is often favoured for its relative simplicity, no motor deficit, and lower perceived risk profile [9]. In contrast, the popliteal block is associated with a longer duration of analgesia and may provide superior postoperative pain control. However, it carries potential drawbacks, including prolonged motor blockade, delayed mobilisation, and increased risk of clinically significant nerve injury compared with distal techniques.

Traditionally, and still in some units, a nerve stimulator is used to approximate the needle placement by eliciting a motor response. This is particularly relevant for popliteal blocks as it is associated with several recognised limitations, including increased failure rates and a heightened risk of vascular puncture, neuropraxia, discomfort caused by involuntary foot twitching, and direct nerve injury, with the potential for iatrogenic foot drop [10]. However, advances in ultrasound‐guided regional anaesthesia have further shaped contemporary practice. Ultrasound guidance is regarded as the gold standard for peripheral nerve blocks, enabling real‐time visualisation of neural and surrounding structures, improving accuracy, and reducing complication rates compared with landmark‐based or nerve stimulator techniques [11]. Although ankle blocks can be performed using anatomical landmarks alone, the integration of ultrasound has enhanced accuracy, reduced neurovascular risk, and improved overall theatre efficiency [9]. However, this is dependent on operator training and competency. Whilst both nerve blocks offer effective analgesia, differences in delivery and analgesic duration may have implications for patient experience, postoperative pain outcomes and opioid use [12].

Despite the widespread use of both ankle and popliteal nerve blocks, there is currently no published evidence evaluating their comparative performance within UK podiatric surgery practice, representing a clear and unaddressed gap in the literature. In the absence of such evidence, variability in clinician preference persists, often driven by individual experience and perceived differences in technical complexity, analgesic profile, and risk. This variation is reflected within our institution, where differences in block selection appear to influence patient outcomes, highlighting ongoing uncertainty regarding the optimal anaesthetic approach for day‐case forefoot surgery. Given the current emphasis on patient‐centred care, enhanced recovery pathways, and service efficiency, the lack of evidence to guide regional anaesthetic choice in podiatric surgery is increasingly difficult to justify. Accordingly, there is a clear need for robust, context‐specific evaluation to support evidence‐based decision‐making and standardisation of clinical practice where appropriate. This service evaluation therefore represents, to the authors' knowledge, the first study to directly compare ultrasound‐guided ankle and popliteal nerve blocks performed as sole anaesthetic techniques, without intravenous sedation or general anaesthesia. The study aims to evaluate differences in analgesic outcomes, functional recovery, patient experience, and operational efficiency. By addressing this critical evidence gap, the findings will inform local clinical practice and provide an initial evidence base to support wider adoption of optimal regional anaesthetic strategies within foot surgery services, ultimately improving the safety, consistency, and quality of patient care.

## 2 Aims and Objectives

The aim and objectives of this project have been formulated to address the evidence gap, specifically the absence of baseline comparative data and the lack of evidence within the UK podiatric surgery context. The primary aim of this project is to investigate the following question:

In adult patients undergoing elective forefoot surgery, how do the ultrasound‐guided ankle and the popliteal nerve blocks compare in terms of postoperative pain intensity, patient experience, and functional recovery in patients undergoing elective forefoot surgery within a UK Podiatric Surgery Unit?

In order to establish a robust and clinically meaningful comparative baseline, the specific, measurable, and achievable objectives of this project are as follows:

1. Onset time: To quantify and compare the time required to achieve complete sensory blockade and, where applicable, the motor blockade, in the ankle and popliteal block groups.
2. Procedural discomfort: To evaluate and compare patient‐reported discomfort using a Visual Analogue Scale (VAS), during the administration of each block.
3. Anaesthetic duration: To measure and compare the duration of postoperative analgesia achieved with the ankle and popliteal blocks.
4. Opioid consumption: To quantify and compare the total opioid medication consumed, measured in Morphine Milligram Equivalents (MME).
5. Functional recovery: To assess and compare postoperative functional recovery using the validated Functional Recovery Index (FRI) (Supporting Information S1: Appendix 1).

## 3 Methods

### 3.1 Study Design and Setting

The study was designed as a prospective comparative service evaluation using a quantitative approach. Clinical outcomes and patient‐reported outcome measures (PROMs) were collected for ultrasound‐guided ankle and popliteal nerve blocks. The evaluation was conducted within a dedicated day‐case podiatric surgical unit in an NHS community trust hospital. The clinical team consisted of two consultant podiatric surgeons, one trainee podiatric surgeon, and one staff podiatrist, all experienced in ultrasound‐guided regional anaesthesia. As multiple operators were involved, variation in block technique was anticipated. However, this reflects the pragmatic, real‐world nature of routine clinical practice.

### 3.2 Population and Recruitment

A prospective consecutive sampling strategy was employed, whereby all eligible patients were recruited sequentially over 6‐months. Inclusion criteria comprised adults aged ≥ 18 years undergoing elective forefoot surgery and able to provide informed consent. To ensure a consistent and clinically meaningful baseline of postoperative pain, inclusion was restricted to patients undergoing at least a first ray osteotomy or fusion. This approach aimed to enhance the sensitivity of the study in detecting differences in analgesic outcomes between nerve block techniques and to minimise the risk of ceiling effects associated with less invasive procedures.

Exclusion criteria included patients with peripheral neuropathy, significant hepatic or renal impairment, known allergy or hypersensitivity to local anaesthetic agents, and pre‐existing chronic pain syndromes requiring regular analgesic use (including long‐term NSAIDs or opioids). Patients undergoing revision surgery or with prior exposure to regional anaesthesia of the foot or ankle were also excluded to minimise bias.

### 3.3 Group Allocation

Group allocation followed the existing clinical workflow. Two surgeons within the service each had a consistent preference for regional anaesthetic technique, with one routinely performing ankle nerve blocks and the other performing popliteal nerve blocks in forefoot surgery involving the first ray. Patients were scheduled sequentially to the next available operating list by the day‐case surgery team, independent of surgeon allocation or anaesthetic technique. This represents a pragmatic, non‐randomised allocation strategy reflective of real‐world service delivery.

Patients were informed pre‐operatively that regional anaesthesia would be administered either at the ankle or popliteal level, without specification of the technique until the day of surgery. As surgical lists were allocated independently by the day‐case coordination team, patients had no prior knowledge of the operating surgeon or nerve block technique.

### 3.4 Standardisation of Interventions

All nerve blocks were performed under ultrasound guidance to ensure accurate needle placement. A standardised local anaesthetic protocol using 0.5% levobupivacaine was applied across both groups. For the popliteal block, 16–18 mL was administered into the sciatic nerve sheath, with an additional 2–4 mL delivered to the saphenous nerve at the anteromedial aspect of the ankle. For the ankle block, a total volume of 10–12 mL was administered to the relevant terminal nerves, including the tibial, deep and superficial peroneal, saphenous, and sural nerves. The sural nerve was selectively excluded where anaesthetic coverage of its dermatome was not required for the planned surgical site. Postoperative opioids included codeine phosphate (15 or 30 mg), dihydrocodeine (30 mg), or oral morphine (5 mL/10 mg). Patients were also instructed to record any over‐the‐counter use of co‐codamol 8/500 mg, with the codeine component incorporated into the calculation. All patients were routinely advised to obtain and use paracetamol and ibuprofen as required. However, consumption of these non‐opioid analgesics was not quantified, as this was outside the predefined objectives of the service evaluation.

### 3.5 Data Collection and Outcome Measures

Data collection focussed on capturing objective measures and PROMs using a bespoke questionnaire developed for this service evaluation (Supporting Information S2: Appendix 2). Data were collected immediately following block administration. Procedural discomfort was assessed using a visual analogue scale (VAS), and block onset time was evaluated at approximately five‐minute intervals using pin‐prick sensory testing across relevant dermatomes. Motor blockade, assessed in the popliteal group, was defined as the inability to actively dorsiflex or plantarflex the ankle.

Postoperatively, the duration of analgesia was defined as the time from block completion to the patient's report of normal sensation. To allow differentiation between acute and prolonged analgesic requirements, opioid consumption was categorised into three postoperative periods: Day 0–3, Day 4–7, and Day 8–14. Furthermore, opioid consumption was standardised to MME, a standardised measure expressing the analgesic dose of different opioids relative to morphine using conversion factors provided by the Faculty of Pain Medicine [13]. Finally, functional recovery was assessed on postoperative Days 1 and 3 using a validated, modified FRI, with non‐applicable items excluded to reflect the immediate postoperative context and capture recovery during the peak pain period [14, 15].

### 3.6 Data Analysis

Descriptive and inferential statistical analyses were performed using JASP [version 0.95.4], with the threshold for significance set at *p* < 0.05 [16]. Descriptive statistics for continuous variables were summarised as means and standard deviations (SD). Although this study was conducted as a service evaluation, inferential statistics were applied to explore differences between groups and assess the consistency of observed effects within the sample. Normality was assessed using the Shapiro–Wilk test, and homogeneity of variances using Levene's test. For between‐group comparisons, independent sample t‐tests were used for normally distributed data, with Welch's *t*‐test applied where variances were unequal. The Mann–Whitney *U* test was used for non‐normally distributed data. Motor onset time was analysed descriptively within the popliteal group only. Longitudinal outcomes, including postoperative pain (VAS), opioid consumption (MME), and functional recovery scores, were analysed using two‐way mixed‐design ANOVA, with block type (ankle vs. popliteal) as the between‐subjects factor and time as the within‐subjects factor. Where significant main effects or interactions were identified, post hoc pairwise comparisons were performed using Holm–Bonferroni correction to adjust for multiple testing.

### 3.7 Ethical Approval

Both regional anaesthetic techniques formed part of routine clinical practice for eligible patients undergoing elective forefoot surgery. This project was conducted as a service evaluation. Therefore, formal Research Ethics Committee (REC) approval and submission via the Integrated Research Application System (IRAS) were not required, in accordance with Health Research Authority (HRA) guidance. Patients provided written consent for the surgical procedure and additional consent for anonymised outcome data to be used for service evaluation purposes. Local governance approval was obtained from the Research and Development department prior to commencement.

## 4 Results

A total of 23 patients met the eligibility criteria during the recruitment period. Of these, 17 patients were included in the final analysis (ankle *n* = 9; popliteal *n* = 8). Table 1 shows the baseline patient characteristics of the included patients. Six patients were excluded from the analysis. Four exclusions were due to non‐response to postoperative data collection, one due to protocol deviation, and one due to complete block failure requiring rescheduling under general anaesthesia. Notably, one of the non‐responding patients from the popliteal block group developed postoperative neuropraxia, which persisted for approximately 12 weeks before complete resolution and required pharmacological management with amitriptyline 10 mg. The full dataset is available as supplementary material.

**TABLE 1:** Patient baseline characteristics (*N* = 17).

Sex	
Female, *n* (%)	15 (88.24)
Male, *n* (%)	2 (11.76)
Mean age, years (SD)	63.12 (11.70)
Surgical procedures, ankle group, *n* = 9	
Metaphyseal extra‐articular transverse akin	6
First MTPJ fusion	2
MIS first MTPJ fusion	1
Surgical procedures, popliteal group, *n* = 8	
Scarf and akin osteotomies	3
First MTPJ fusion	5^a^
Weil's osteotomy	1^a^
Mean motor onset time after popliteal block, minutes (SD)	23.29 (18.05)

Abbreviations: MTPJ, metatarsophalangeal joint; SD, standard deviation.

^a^ *n* = 1 patient underwent multiple surgical procedures (first MTPJ fusion and second and third Weil's osteotomy).

### 4.1 Sensory and Motor Onset

**FIGURE 1:**
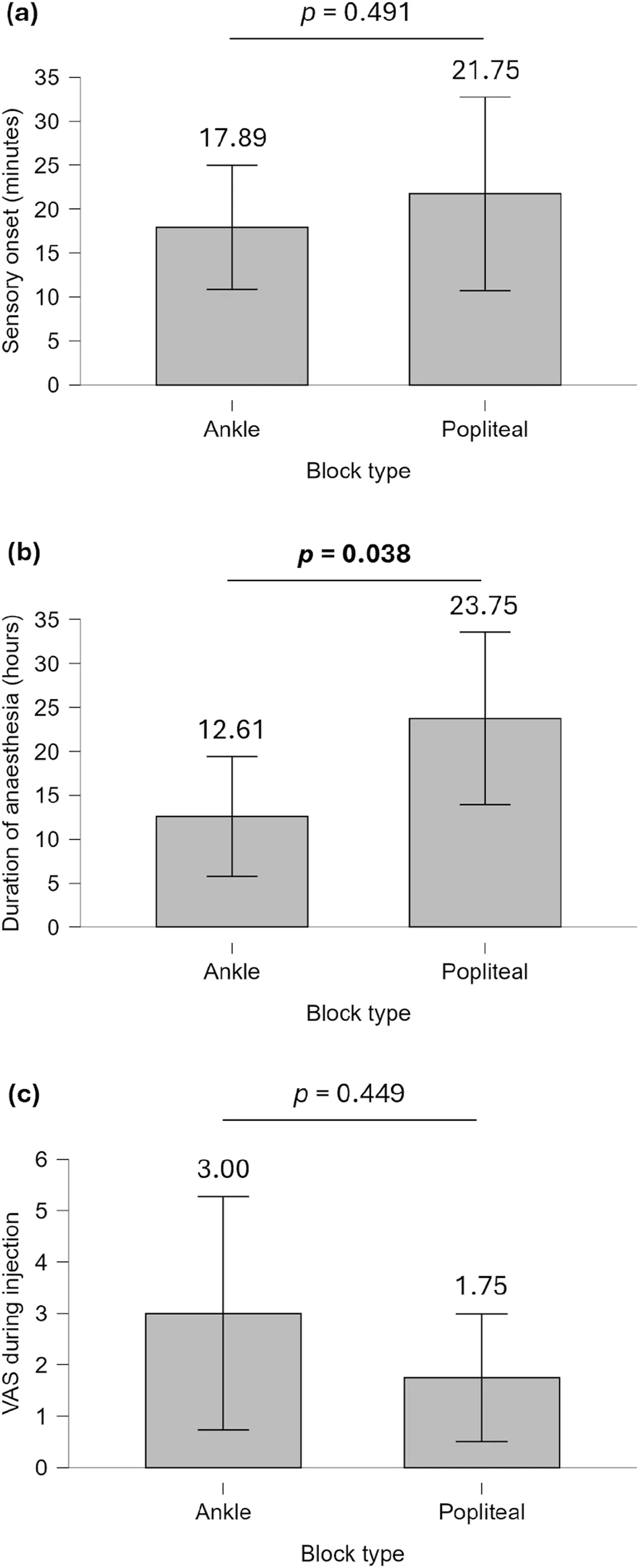
Between‐group comparison of (a) sensory onset, (b) duration of anaesthesia, and (c) procedural discomfort.

Normal distribution (*p* = 0.304) and equality of variance (*p* = 0.204) were confirmed for sensory onset. An independent sample *t*‐test showed no statistically significant difference between groups (*p* = 0.491, Table 2). The ankle block group achieved complete sensory blockade at a mean (SD) of 17.89 (9.19) minutes, compared with 21.75 (13.23) minutes in the popliteal block group (Figure 1a), representing a small effect size (Cohen's *d* = −0.34). The mean (SD) onset time for motor blockade in the popliteal block group was 23.29 (18.05) minutes (Table 2). One patient in this group did not develop motor blockade but achieved complete sensory blockade and proceeded with surgery as planned.

**TABLE 2:** Block characteristics of sensory onset, duration of anaesthesia and procedural discomfort.

	Ankle (*n* = 9)	Popliteal (*n* = 8)	Effect size	*p*
Mean sensory onset, minutes (SD)	17.89 (9.19)	21.75 (13.23)	−0.34^a^	0.491^a^
Mean duration of anaesthesia, hours (SD)	12.61 (8.87)	23.75 (11.77)	0.61^b^	**0.038** ^b^
Mean VAS during injection (SD)	3.00 (2.96)	1.75 (1.49)	−0.22^b^	0.449^b^

*Note:* Bolded values indicate statistical significance.

Abbreviations: SD, standard deviation; VAS, visual analogue scale.

^a^ Cohen's *d*.

^b^ Rank‐biserial correlation.

### 4.2 Duration of Anaesthesia

The Shapiro‐Wilk test indicated a deviation from normality (*p* = 0.040). Consequently, the Mann–Whitney *U* test was utilised, identifying a statistically significant difference (*U* = 14.0, *p* = 0.038, Table 2). The mean (SD) duration of analgesia was longer in the popliteal block group at 23.75 (11.77) hours compared with 12.61 (8.87) hours in the ankle block group (Figure 1b). The associated effect size was large (rank‐biserial correlation = 0.61), suggesting a substantial difference in analgesic duration between techniques.

### 4.3 Procedural Discomfort

Patient‐reported discomfort during block administration, measured using VAS scores, demonstrated a non‐normal distribution (*p* = 0.002, Table 2). Accordingly, a Mann–Whitney *U* test was performed and identified no statistically significant difference between groups (*U* = 44.0, *p* = 0.449). Mean (SD) VAS scores were 3.00 (2.96) in the ankle block group and 1.75 (1.49) in the popliteal block group (Figure 1c). The small effect size (rank‐biserial correlation = −0.22) indicates that both techniques were well tolerated and that block selection did not significantly impact procedural discomfort.

### 4.4 Postoperative Pain

**FIGURE 2:**
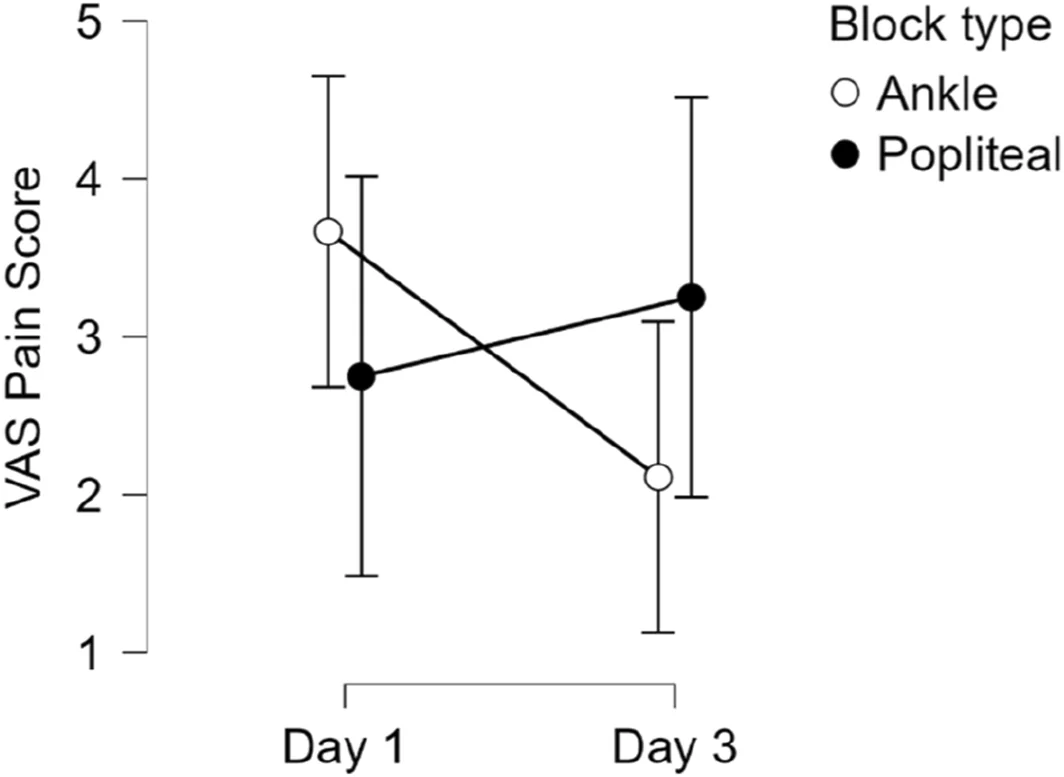
Postoperative pain scores on Day 1 and Day 3.

ANOVA was conducted to assess changes in postoperative pain from Day 1 to Day 3 (Table 3). There were no significant main effects of time (*p* = 0.288) or block type (*p* = 0.907). However, a statistically significant interaction between time and block type was observed [F(1, 15) = 4.61, *p* = 0.049], indicating differential changes in pain over time between groups. Specifically, patients in the ankle block group reported higher VAS scores on Day 1, at a mean (SD) of 3.67 (2.18), which decreased to 2.11 (1.27) by Day 3 (Table 4). In contrast, the popliteal block group reported lower initial pain scores on Day 1 at 2.75 (2.44), followed by an increase by Day 3 at 3.25 (2.61) (Figure 2).

**TABLE 3:** ANOVA summary table: VAS pain scores on Day 1 versus Day 3.

Effect	df	*F*	*p*
Time (day 1 vs. day 3)	1, 15	1.216	0.288
Block type (ankle vs. popliteal)	1, 15	0.014	0.907
Interaction (time × block type)	1, 15	4.610	**0.049**

*Note:* Bolded values indicate statistical significance.

Abbreviation: df, degrees of freedom.

**TABLE 4:** VAS pain scores on Day 1 and Day 3.

	Ankle (*n* = 9)	Popliteal (*n* = 8)
VAS pain score at day 1 (SD)	3.67 (2.18)	2.75 (2.44)
VAS pain score at day 3 (SD)	2.11 (1.27)	3.25 (2.61)

Abbreviations: SD, standard deviation; VAS, visual analogue scale.

### 4.5 Opioid Consumption

**FIGURE 3:**
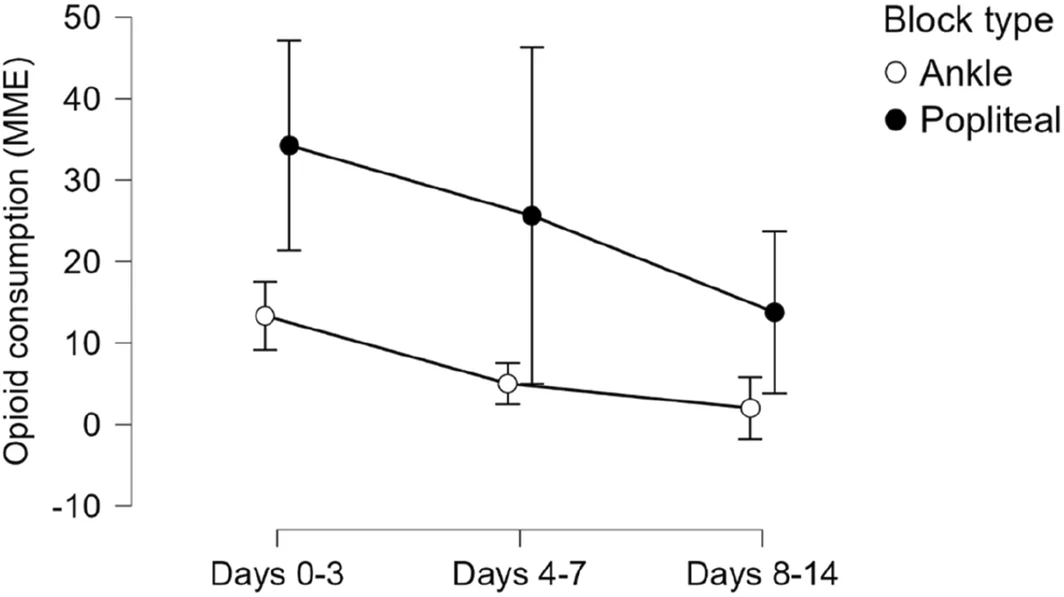
Opioid consumption between Days 0 and 14.

ANOVA was conducted to assess opioid consumption across three postoperative intervals: Days 0–3, Days 4–7, and Days 8–14 (Table 5). Mauchly's test indicated a violation of the sphericity assumption (*χ* ^2^ = 28.17, *p* < 0.001). Therefore, Greenhouse‐Geisser‐corrected degrees of freedom were applied. A statistically significant main effect of time was observed [*F*(1.25, 18.76) = 6.51, *p* = 0.015], reflecting a progressive reduction in opioid requirements over the 14‐day postoperative period. Holm‐corrected post hoc analyses demonstrated that this reduction was primarily driven by a significant decrease in consumption between the acute postoperative phase (Days 0–3) and the late interval (Days 8–14; *p*_holm_ = 0.003). In contrast, there was no significant main effect for block type (*F* (1, 15) = 2.00, *p*_holm_ = 0.178) and no significant interaction between time and block type (*F* (1.25, 18.76) = 0.70, *p*_holm_ = 0.446). The popliteal group exhibited higher mean opioid consumption across all intervals (Figure 3). For example, Days 0–3: mean (SD) 34.25 (29.58) MME in the popliteal group versus 13.33 (10.19) MME in the ankle group (Table 6).

**TABLE 5:** ANOVA summary table: Opioid consumption between days 0 and 14.

Effect	df	*F*	*p*
Time (intervals 1, 2, 3)	1.25, 18.76	6.506	**0.015**
Block type (ankle vs. popliteal)	1, 15	1.998	0.178
Interaction (time × block type)	1.25, 18.76	0.696	0.446

*Note:* Degrees of freedom (df) for within‐subjects’ effects were adjusted using Greenhouse‐Geisser corrections (*ε* = 0.625) as Mauchly's test indicated the assumption of sphericity was violated (*p* < 0.05). Bolded values indicate statistical significance.

**TABLE 6:** Opioid consumption on Days 0–3, Days 4–7 and Days 8–14.

	Ankle (*n* = 9)	Popliteal (*n* = 8)
Opioid consumption on days 0–3, MME (SD)	13.33 (10.19)	34.25 (29.58)
Opioid consumption on days 4–7, MME (SD)	5.00 (10.17)	25.63 (55.71)
Opioid consumption on days 8–14, MME (SD)	2.00 (6.00)	13.75 (27.43)

Abbreviation: SD, standard deviation.

### 4.6 Functional Recovery

**FIGURE 4:**
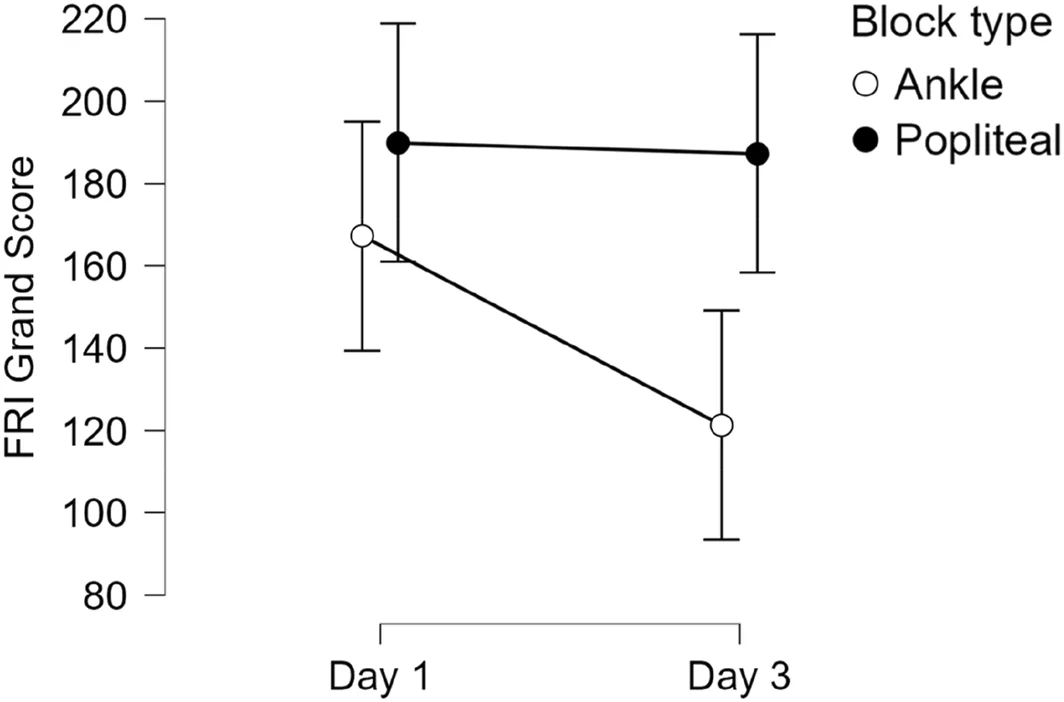
Functional recovery on Day 1 and Day 3. FRI, functional recovery index.

FRI grand scores were analysed using a two‐way ANOVA (Table 7). The assumption of normality was satisfied (*p* > 0.05). No statistically significant main effect of block type was observed [*F* (1,15) = 0.924, *p* = 0.352], indicating comparable overall functional outcomes across time points. Two effects approached statistical significance. First, there was a trend towards functional improvement over time across the entire cohort [*F* (1,15) = 3.962, *p* = 0.065], suggesting recovery between Day 1 and Day 3. Second, the time‐by‐block type interaction also approached significance [*F* (1,15) = 3.151, *p* = 0.096], suggesting a potential divergence in early postoperative recovery trajectories between the two block techniques. Descriptive data revealed a clearer clinical pattern (Table 8). The ankle block group demonstrated an improvement in functional scores, with mean (SD) FRI grand scores decreasing from 167.20 (92.03) on Day 1–121.30 (100.59) on Day 3 (Figure 4). In contrast, the popliteal block group showed minimal change over the same period, with scores of 189.90 (106.78) on Day 1 and 187.30 (92.62) on Day 3. Holm‐corrected post hoc analyses indicated that improvement in the ankle group was the only contrast approaching significance (*p*_holm_ = 0.090), whereas no meaningful change was observed in the popliteal group (*p*_holm_ = 1.000).

**TABLE 7:** ANOVA summary table: Functional recovery on Day 1 and Day 3.

Effect	df	*F*	*p*
Time (day 1 vs. day 3)	1, 15	3.962	0.065
Block type (ankle vs. popliteal)	1, 15	0.924	0.352
Interaction (time × block type)	1, 15	3.151	0.096

**TABLE 8:** Functional recovery on Day 1 and Day 3.

	Ankle (*n* = 9)	Popliteal (*n* = 8)
FRI grand score at day 1 (SD)	167.20 (92.03)	189.90 (106.78)
FRI grand score at day 3 (SD)	121.30 (100.59)	187.30 (92.62)

Abbreviations: FRI, functional recovery index; SD, standard deviation.

## 5 Discussion

This service evaluation contributes to an underrepresented area within the UK literature by directly comparing ankle and popliteal nerve blocks within podiatric surgical practice. Historically, Coutts [17] reported the use of podiatrist‐administered popliteal nerve blocks using ultrasound or nerve stimulation in patients undergoing day‐case hallux valgus surgery, providing early evidence for podiatrist‐led regional anaesthesia in this setting. More recently, Kang et al. [18] retrospectively evaluated 101 consecutive patients undergoing elective foot surgery within a UK podiatric surgery department and reported a 93.1% success rate for ultrasound‐guided popliteal blocks, alongside prolonged postoperative analgesia, good patient tolerance, and no serious complications. These findings provide evidence that ultrasound‐guided popliteal nerve blockade can be effectively delivered within podiatric surgical practice. However, as a retrospective case series without a comparator group, the study demonstrates feasibility and clinical effectiveness rather than comparative superiority. Together, these studies support the established role of podiatrist‐delivered ultrasound‐guided regional anaesthesia within UK podiatric surgery, while highlighting the continued need for prospective comparative research to determine the relative clinical characteristics and outcomes of different block techniques.

Moreover, with the increasing number of podiatrists annotated as independent prescribers, there is a clear pathway to evolving the standard of care [19]. These baseline data enable future exploration of pharmacological adjuvants, such as liposomal bupivacaine and dexamethasone, designed to extend the analgesic window in podiatric surgery in the UK. Liposomal bupivacaine is a prolonged‐release formulation in which bupivacaine is encapsulated within multivesicular liposomes, allowing gradual drug release over time and thereby extending the duration of postoperative analgesia compared with conventional bupivacaine [20]. In a prospective cohort study (20 vs. 20), adding liposomal bupivacaine as an adjunct to the ankle block in forefoot surgery significantly reduced the mean number of narcotic pills consumed on postoperative day 1 (1.4 vs. 3.6, *p* = 0.002) and day 2 (1.8 vs. 3.6, *p* = 0.021) [21]. However, daily pain scores were lower in the liposomal bupivacaine group than in the control group, but the difference was not statistically significant. Similar findings have been observed in a prospective RCT, in which perineural popliteal block with liposomal bupivacaine in foot and ankle surgery led to less opioid consumption postoperatively and a longer block duration compared with local anaesthetic alone or surgeon field block with liposomal bupivacaine [22]. A Phase 3 RCT corroborated these findings, demonstrating that adding liposomal bupivacaine to the popliteal nerve block reduced pain and opioid consumption over 4 days postoperatively versus bupivacaine hydrochloride alone and increased the proportion of opioid‐free patients (24.4% vs. 6%) with similar adverse events [23]. Regarding dexamethasone, which can be administered either perineurally or intravenously, RCTs have shown it to be useful as an adjuvant to popliteal, saphenous, or ankle blocks, prolonging analgesia and reducing opioid requirements [24, 25, 26, 27]. When comparing intravenous and perineural dexamethasone, a meta‐analysis found that perineural dexamethasone increased analgesia by 2.7 hours on average, with no differences in opioids or adverse effects [28]. However, the authors judged this unlikely to be clinically relevant and emphasised that perineural is off‐label. Notably, all these studies were conducted in conjunction with general anaesthesia and anaesthetist‐delivered regional techniques, reflecting conventional perioperative models within orthopaedic foot and ankle surgery. In contrast, podiatric surgery in the UK uniquely integrates regional anaesthesia within the surgical role itself, frequently delivering regional nerve blocks as the sole anaesthetic modality. While not intended to replace the role of anaesthetists, this approach highlights a complementary pathway for elective care delivery that may enhance patient safety, improve efficiency, and support efforts to reduce surgical waiting lists across the NHS.

From the perspective of a UK podiatric surgery unit, these findings serve as a benchmark for regional anaesthesia, validating the clinical scope and procedural effectiveness of the speciality. Regarding sensory onset, the data from this evaluation showed a high degree of equivalence, with no statistically significant difference between the ankle and popliteal cohorts (*p* = 0.491). Both techniques achieved complete sensory blockade within comparable timeframes. In contrast, the popliteal block demonstrated a significantly longer duration of anaesthesia, providing a mean (SD) duration of effect of 23.75 (11.77) hours, compared with 12.61 (8.87) hours for the ankle block (*p* = 0.038). This near doubling of analgesic duration is consistent with the anatomical advantages of a more proximal nerve blockade and supports its capacity to prolong primary postoperative analgesia prior to the return of normal sensation [29]. Procedural discomfort was comparable between techniques. Pain during block administration was low overall, and no statistically significant difference in VAS scores was identified between groups (*p* = 0.449). Although the ankle group reported a slightly higher mean (SD) VAS score of 3.00 (2.96) compared with 1.75 (1.49) in the popliteal group, the small effect size indicates no clinically meaningful difference in procedural discomfort (rank‐biserial correlation = −0.22). Overall, patient comfort during regional block administration appears comparable between techniques and is more likely influenced by individual patient factors, such as anxiety, pain sensitivity, and anatomical variability, rather than by block type alone. The slightly higher mean discomfort observed in the ankle block group may reflect the requirement for multiple needle insertions to achieve complete blockade, typically involving four to five injections, in contrast to the fewer injection sites required for a popliteal block. However, this did not translate into a statistically or clinically significant difference.

Analysis of postoperative pain trajectories identified a statistically significant interaction between time and block type (*p* = 0.049), indicating divergent recovery patterns. Patients receiving an ankle block reported higher pain scores on Day 1 followed by a reduction by Day 3. In contrast, those in the popliteal group experienced lower initial pain scores with a subsequent increase by Day 3. This pattern suggests that while the popliteal block effectively delays the onset of postoperative pain, it may be associated with a more abrupt analgesic transition once the block resolves, in contrast to the more gradual pain course observed with the ankle block. However, this finding must be interpreted in the context of the operative technique. In the ankle block cohort, 7 of 8 procedures used minimal incision surgery (MIS) techniques. In contrast, all patients in the popliteal group underwent conventional open surgery. MIS approaches are associated with reduced soft‐tissue disruption and inflammatory burden [30]. Therefore, while speculative, it is plausible that the more favourable pain trajectory observed in the ankle group reflects, at least in part, the influence of the surgical approach rather than the regional block alone. Future studies should prospectively control for operative technique or stratify analyses by MIS versus open procedures to isolate the independent contributions of block type and surgical invasiveness to postoperative pain trajectories. Nevertheless, postoperative pain remained consistently low across both cohorts, with mean VAS scores within the mild range at all time points, suggesting that both regional techniques maintain clinically acceptable analgesia throughout the early recovery phase.

Total opioid consumption did not significantly differ between groups across the 14‐day postoperative period (*p* = 0.178). A significant main effect of time was observed for the cohort as a whole (*p* = 0.015), reflecting a general reduction in opioid requirements as recovery progressed. Despite prolonged block duration, no statistically significant reduction in opioid consumption was observed in the popliteal group. The absence of between‐group significance is likely attributable to substantial inter‐individual variability within the popliteal cohort, with standard deviations exceeding the corresponding means. For instance, Days 4–7 at mean (SD) 25.63 (55.71) MME. This degree of dispersion suggests heterogeneous postoperative opioid requirements, which may have attenuated the ability to detect group‐level differences. Finally, functional recovery patterns measured using the FRI broadly mirrored pain trajectories although the interaction effect did not reach statistical significance (*p* = 0.096). The ankle block group demonstrated a clear trend towards improved functional scores between Day 1 and Day 3, whereas the popliteal group showed minimal change over the same period. This pattern reflects the rebound phenomenon discussed in relation to pain. As ankle blocks typically resolve earlier than popliteal blocks, patients may reach a threshold of functional readiness sooner. By Day 3, those in the ankle group were likely to have progressed beyond the peak pain phase and regained sufficient motor control to support basic mobilisation. In contrast, the prolonged sensory and motor effects associated with popliteal blocks may delay this transition, leading to a deferred analgesic decline and residual motor impairment that constrain early functional gains. Although falls were not assessed within this evaluation, temporary motor impairment following popliteal blockade may warrant consideration when planning postoperative mobilisation, particularly in the older patient cohort and those with baseline mobility impairment.

Several limitations must be considered when interpreting these findings. As this study was undertaken as a prospective service evaluation, no formal a priori sample size calculation was performed. Consequently, the sample size was determined by the number of eligible patients presenting during the evaluation period. Although this pragmatic approach reflects real‐world practice, the sample size (*n* = 17) limited statistical power and increased the risk of Type II error. This limitation is amplified by the high inter‐individual variability observed across several outcomes, particularly opioid consumption and FRI scores, suggesting that patient‐specific factors may have exerted a greater influence on outcomes than block type alone. Furthermore, there was a notable mismatch in surgical procedures between groups, with 7 of 8 patients in the ankle block cohort undergoing MIS techniques. Because less invasive procedures inherently result in reduced tissue trauma, this surgical imbalance represents a significant confounder when comparing pain trajectories and analgesic efficacy. Additionally, the evaluation was also vulnerable to observer and performance bias, as clinicians administering the blocks were responsible for recording onset times. Although reflective of real‐world practice, the absence of assessor blinding introduces the potential for subconscious expectation bias. This risk is compounded by inter‐observer variability in the operational definition of block onset. Despite prior briefings, the assessment of sensory and motor block onset is inherently subjective, and inter‐clinician variation in judgement may have introduced measurement error and reduced the reliability of onset time measurements.

## 6 Conclusion

This service evaluation demonstrates that both ankle and popliteal nerve blocks provide effective perioperative anaesthesia for elective forefoot surgery, with overall outcomes influenced more by the characteristics of each technique than by differences in sensory onset. While onset times were comparable, popliteal blocks produced a significantly longer duration of postoperative analgesia, effectively extending the pain‐free period when compared with ankle blocks. In contrast, ankle blocks were associated with a more favourable early functional recovery profile, likely reflecting the absence of prolonged motor blockade and the delayed rebound pain observed in the popliteal cohort. These findings will inform clinical practice by developing a patient‐centred, risk‐stratified block selection protocol. In addition, standardising ultrasound guidance for proximal nerve blocks may enhance safety and procedural efficiency by reducing the risk of neuropraxia and transient motor deficits, which remain key considerations in popliteal block administration.

Importantly, this evaluation establishes a baseline for UK podiatric surgery, demonstrating that advanced regional anaesthetic techniques are firmly within the speciality's scope of practice. The findings provide a platform for future prospective research, including RCTs investigating pharmacological adjuvants such as dexamethasone or liposomal bupivacaine in the context of UK podiatric surgery. Incorporating these may help address the analgesic gap identified in this study, facilitating a smoother transition from operative anaesthesia to postoperative recovery and further advancing patient‐centred, evidence‐based podiatric surgical care.

## Author Contributions

**Yang Sern Kang:** conceptualization, data curation, formal analysis, investigation, methodology, project administration, visualization, writing – original draft, writing – review and editing. **Tommy Chan:** investigation, writing – review and editing. **Robert Morley:** investigation, writing – review and editing.

## Funding

The authors have nothing to report.

## Ethics Statement

Both regional anaesthetic techniques form part of routine clinical practice for eligible patients undergoing elective forefoot surgery. This project was conducted as a service evaluation. Therefore, formal Research Ethics Committee (REC) approval and submission via the Integrated Research Application System (IRAS) were not required, in accordance with Health Research Authority (HRA) guidance. Patients provided written consent for the surgical procedure and additional consent for anonymised outcome data to be used for service evaluation purposes. Local governance approval was obtained from the Research and Development department prior to commencement.

## Conflicts of Interest

The authors declare no conflicts of interest.

## Supporting information


Supporting Information S1

Supporting Information S2

Supporting Information S3

## Data Availability

The data that supports the findings of this study are available in the supplementary material of this article.
