# Supplementary material for: A Prospective Comparison of Ultrasound‐Guided Ankle Versus Popliteal Nerve Blocks for Elective Forefoot Surgery in a UK Podiatric Surgery Unit
Source: J Foot Ankle Res. 2026 Sep 23;19(3):e70219. doi: 10.1002/jfa2.70219 (PMC13601650; doi:10.1002/jfa2.70219)
Supplement: Supplementary file 1 [file JFA2-19-e70219-s002.docx]

**Appendix 1. Functional Recovery Index (FRI)**

**Factor 1: pain and social activity**

Have you experienced difficulty with the following since your surgery:

No difficulty at all ———————— Extreme difficulty

**0	1	2	3	4	5	6	7	8	9	10	NA**

1 Returning to work

2 Visiting friends, relatives

3 Taking care of other family member

4 Driving a car

5 Moderate exercise, such as moving a table or pushing a vacuum cleaner

6 Lifting or carrying groceries

7 Since your surgery, has pain been a problem?

**Factor 2: lower limb activity**

Since your surgery, have you experienced difficulty in:

No difficulty at all ———————— Extreme difficulty

**0	1	2	3	4	5	6	7	8	9	10	NA**

1 Walking indoors or around the house

2 Moving in/out of a chair

3 Climbing stairs

4 Bending, kneeling, or stooping

**Factor 3: general physical activity**

Since your surgery, have you experienced difficulty in:

No difficulty at all ———————— Extreme difficulty

**0	1	2	3	4	5	6	7	8	9	10	NA**

1 Taking a bath/shower/cleaning yourself

2 Dressing

3 Lying down

**Grand score = (total of all the scores) × 14/number of answered questions = ____________________**

If patients do not normally perform such activities, e.g., driving, choose not applicable (NA). If patients were instructed by surgeons not to perform any of the activities, choose NA.
