# Supplementary material for: A Prospective Comparison of Ultrasound‐Guided Ankle Versus Popliteal Nerve Blocks for Elective Forefoot Surgery in a UK Podiatric Surgery Unit
Source: J Foot Ankle Res. 2026 Sep 23;19(3):e70219. doi: 10.1002/jfa2.70219 (PMC13601650; doi:10.1002/jfa2.70219)
Supplement: Supplementary file 2 [file JFA2-19-e70219-s003.docx]

Appendix 2: Questionnaire

Date of surgery:

Block type:

□ Ankle	□ Popliteal

Administered by:

**Ankle versus Popliteal Nerve Blocks in Elective Forefoot Surgery - Patient Questionnaire**

SECTION A: Anaesthetic Effect

1. Time taken for anaesthesia *(To be completed by the clinician)*

Time administered: ____________

Onset time (sensory): ______ minutes *(Assess via pinprick/sharp test)*

Total volume of anaesthetic administered: _____mL

Top up required? _____mL *(Leave blank if not required)*

Administered by: ______

***(Q2 – Q6 below to be completed by the patient)***

2. How long did the numbness last after the surgery?

Please estimate the time in hours until normal sensation returned:  ______ hours

Section B: Pain Assessment (VAS – Visual Analogue Scale)

**Please circle on the number that best represents your level of pain**

3. How much pain did you experience during administration of the anaesthetic injection(s)?

SECTION C: Postoperative Pain Relief Medication

4. Did you take any opioid-based pain relief medication after surgery (e.g., dihydrocodeine, codeine phosphate, tramadol, morphine)?

☐ Yes 	☐ No

5. If yes, how many opioid tablets/capsules/solution did you take on:

(Please estimate as best as you can and leave blank if not taken)

*Time Point*	*Approx. total number of opioid doses taken*

Post-op Day 0	______ tablets/doses/mL

Post-op Day 1	______ tablets/doses/mL

Post-op Day 2	______ tablets/doses/mL

Post-op Day 3	______ tablets/doses/mL

Post-op Day 4	______ tablets/doses/mL

Post-op Day 5	______ tablets/doses/mL

Post-op Day 6	______ tablets/doses/mL

Post-op Day 7	______ tablets/doses/mL

Post-op Day 8	______ tablets/doses/mL

Post-op Day 9	______ tablets/doses/mL

Post-op Day 10	______ tablets/doses/mL

Post-op Day 11	______ tablets/doses/mL

Post-op Day 12	______ tablets/doses/mL

Post-op Day 13	______ tablets/doses/mL

Post-op Day 14	______ tablets/doses/mL

SECTION D: Adapted Functional Recovery Index (FRI)

6. Please circle the number that best represents how difficult the following were after your surgery.

**DAY 1 (~24 hours)**

***Factor 1: pain***

Have you experienced difficulty with pain?

***Factor 2: lower limb activity***

Have you experienced difficulty in:

a) Walking indoors or around the house

b) Moving in/out of a chair

c) Climbing stairs

d) Bending, kneeling, or stooping

***Factor 3: general physical activity***

Have you experienced difficulty in:

a) Taking a bath/shower/cleaning yourself

b) Dressing

c) Lying down

Grand score = [sum of all the scores / 80] x 100% **(TO BE CALCULATED BY CLINICIAN)**

=______%

**DAY 3 (~72 hours)**

***Factor 1: pain and social activity***

Have you experienced difficulty with pain?

***Factor 2: lower limb activity***

Have you experienced difficulty in:

e) Walking indoors or around the house

f) Moving in/out of a chair

g) Climbing stairs

h) Bending, kneeling, or stooping

***Factor 3: general physical activity***

Have you experienced difficulty in:

d) Taking a bath/shower/cleaning yourself

e) Dressing

f) Lying down

Grand score = [sum of all the scores / 80] x 100% **(TO BE CALCULATED BY CLINICIAN)**

=______%

**End of Questionnaire**

Please remember to bring this completed questionnaire with you to your post-op follow-up appointment.
